# Breast cancer organoids from a patient with giant papillary carcinoma as a high-fidelity model

**DOI:** 10.1186/s12935-020-01171-5

**Published:** 2020-03-18

**Authors:** Xuelu Li, Bo Pan, Xiaoqing Song, Ning Li, Dongyi Zhao, Man Li, Zuowei Zhao

**Affiliations:** 1 Department of Oncology & Department of Breast Surgery, The Second Hospital of Dalian Medical University, Dalian, 116023 China; 2 Department of Pathology, First Affiliated Hospital and College of Basic Medical Sciences, China Medical University, Shenyang, 110001 China; 3 Department of Foreign Language, Dalian Medical University, Dalian, 116000 China

**Keywords:** Papillary carcinoma, Organoid culture, Individualized therapy, Drug sensitivity test, Breast cancer

## Abstract

**Background:**

Papillary carcinoma is an uncommon type of breast cancer. Additionally, patients with huge breast papillary carcinoma are extremely rare in clinical practice. To improve therapeutic effect on such patients, it is urgent to explore biologically and clinically relevant models of the disease to discover effective drugs.

**Methods:**

We collected surgical tumor specimens from a 63-year-old Chinese woman who has been diagnosed breast papillary carcinoma. The tumor was more than 15 cm in diameter, and applied to establish patient-derived papillary carcinoma organoids that could continuously propagate for more than 6 months.

**Results:**

The papillary carcinoma organoids matched the histological characteristics of orginal tumor by H&E staining identification, and maintained the expression of the breast cancer biomarkers by IHC, including estrogen receptor (ER), progesterone receptor (PR), human epidermal growth factor receptor (HER2) and antigen Ki-67 (Ki67). In addition, we performed a 3-D drug screening to examine the effects of endocrine drugs (Fulvestrant, Tamoxifen) and targeted therapy drugs (Palbociclib, Everolimus, BKM120) on breast papillary carcinoma in the mimic in vivo environment. The drug sensitivities of our breast papillary carcinoma organoids were investigated as follows, Fulvestrant (IC_50_ 0.275 μmol), Palbociclib (IC_50_ 2.21 μmol), BKM120 (IC_50_ 3.81 μmol), Everolimus (IC_50_ 4.45 μmol), Tamoxifen (IC_50_ 19.13 μmol).

**Conclusions:**

These results showed that an effective organoid platform for 3-D in vitro culture of breast cancer organoids from patients with breast papillary carcinoma could be used to identify possible treatments, and might be commonly applied to explore clinicopathological characteristics of breast papillary carcinoma.

## Background

Breast cancer is the most commonly diagnosed cancer (11.6%) and the leading cause of cancer death (6.6%) among females globally [1]. Papillary lesions of the breast initiate within the ducto-lobular system supported by fibrovascular cores, and comprise a heterogeneous group of neoplasms, including (a) intraductal papilloma, (b) papilloma with atypical ductal hyperplasia, (c) papilloma with ductal carcinoma in situ, and (d) papillary carcinoma [2, 3]. Papillary carcinoma is an uncommon type of breast cancers with a better prognosis, accounting for 0.5–1% of breast cancers, and contains encapsulated and solid types [2–4]. Troxell et al. [5] investigated the molecular differences between benign and malignant papillomas. They found that breast papillary carcinomas showed a lower frequency of mutations (PIK3CA, AKT1, NRAS) compared with benign papillomas. By using sanger sequencing, Lozada et al. [6] validated that papillary carcinomas lacked IDH2 mutations, while one out of ten harboured PIK3CA-H1047R mutation. Several studies [7, 8] demonstrated that breast papillary carcinomas displayed a luminal phenotype and a lower prevalence of lymphovascular invasion, as well as lymph node metastasis, which provided a rationale for good clinical outcome. However, only few studies showed molecular characteristics of breast papillary carcinoma. Therefore, we urgently need to develop a feasible and reliable model to study the pathogenesis of papillary carcinoma.

In most studies, researchers use cell lines or animals as research subjects [9]. Recent study demonstrates that methyl-β-cyclodextrin (MCD) efficiently enhances the toxic effects of Doxorubicin (DOX) through depleting membrane cholesterol. Combination of low dose of DOX with suboptimal dose of MCD can serve as a potential strategy to minimize side effects, thereby enhancing the therapeutic efficacy of DOX in breast and HCC cells [10]. Muhammad et al. demonstrated that bitter melon extract treatment inhibits breast tumor growth. Importantly, the anti-tumor activity is partially mediated by induction of autophagy and modulation of the AMPK/mTOR pathway by using advanced preclinical model [11].

Although breast cancer has been studied extensively, we still crucially need a new preclinical disease research model to direct individualized treatment of patients. The term “organoid” reflects the ability of culture conditions to drive cells to self-organize themselves into structures that mimic the architecture of the organ from which they were derived [12]. The microenvironment in organoids resembles the original tumor microenvironment more accurately than that in traditional 2D cultures, which should have a higher success rate than previous primary cultures [13]. Patient-derived tumor organoids (PDTOs) not only recapitulate histological and genetic features of original tumors, but also allow high-throughput drug screening and potentially facilitate personalized therapy [13]. So far, long-term organoid cultures can be established from a variety of cancers, including colorectal [14], gastrointestinal [15], pancreas [16], liver [17], prostate [18], bladder [19], lung [20], and cervix [21] cancer etc. Breast cancer organoids have also emerged as a useful pre-clinical model for maintaining sufficient fidelity regarding the histology, transcriptome and genome [13].

In this study, we describe the case of a 63-year-old Chinese woman with giant breast papillary carcinoma. We obtained breast cancer tissues from this patient who underwent the modified radical mastectomy. The organoid culture technique was used to cultivate patient-derived breast cancer cells. To the best of our knowledge, this is the first report that presents the establishment of papillary carcinoma patient-derived organoids. Most importantly, this confirmed that our 3-D in vitro model was a reliable platform for identifying individual treatment options.

## Materials and methods

### Patient and sample collection

The tumor sample was obtained from the papillary carcinoma patient at the time of surgery. The breast cancer tissue was cut into pieces of soybean size. Two random pieces were snap frozen with liquid nitrogen and stored at − 80 °C for DNA and RNA sequencing. Two random pieces were fixed in formalin for immunohistochemistry. This study was approved by the ethical committees of The Second Hospital of Dalian Medical University (Dalian, China). All the procedures were carried out in accordance with the Declaration of Helsinki.

### Immunohistochemistry

To maintain the three-dimensional structure of the organoids, BME-organoid mixture was aspirated from the 24-well plates gently and completely, then it was embedded in Collagen (Biocoat, Corning, NY, USA). Collagen was added to pre-warmed 48-well plates (Nest, Wuxi, Jiangsu, China) and solidified at 37 °C for 2 h. All samples were fixed in 4% paraformaldehyde before embedded in paraffin. The protein expression was assessed following a two-step method. Rabbit anti-ER antibody (1:5), anti-PR (1:100), anti-HER2 (1:1000), and anti-Ki67 (1:200) were purchased from Abcam (Cambridge, MA, UK). Anti-CK5/6 antibody, Anti-p63, Anti-Calponin and the DAB kit were purchased from Zhongshan Goldenbridge Biotechnology Company (Beijing, China).

### Organoid culture

The surgical tissue of breast cancer were placed in cold AdDF+++ [advanced DMEM/F12 (Sigma, Saint Louis, MO, USA) containing 1 × Glutamax (Invitrogen, Carlsbad, CA, USA), 10 mM HEPES (Invitrogen, Carlsbad, CA, USA) and antibiotics (Sigma, Saint Louis, MO, USA)] and shipped to the laboratory within 20 min on ice. The tissue was cut into 3 mm^3^ pieces, washed with 5 mL of AdDF+++ and digested in 3 mL medium containing 2 mg/ml collagenase (Sigma, Saint Louis, MO, USA) at 37 °C for 2 h or more. During digestion, pipetting is applied to promote release of the cells in solution. The digested tissue was suspended in 3 ml AdDF+++, then centrifuged at 1300 rpm. If a red precipitate is formed, red blood cells are lysed in 1 mL of red blood cell lysis buffer (Roche, Basel, Switzerland) for 5 min at room temperature and centrifuged at 1300 rpm after adding 3 ml of AdDF +++. Then the pellet was suspended in 12 mg/ml cold Cultrex growth factor reduced BME type 2 (Trevigen, Gaithersburg, MD, USA). 40 μL of BME-cell suspension droplets were added to a preheated 24-well suspension plate (Corning Incorporated, NY, USA) at 37 °C for 30 min. After gelation was completed, 500 μL of medium was added to each well, containing AdDMEM/F12 medium supplemented with B27 supplement [1×, Invitrogen, Carlsbad, CA, USA], Nicotinamide [5 mM, Sigma, Saint Louis, MO, USA], GlutaMax 100x [1x, Invitrogen, Carlsbad, CA, USA], Hepes [10 mM, Invitrogen, Carlsbad, CA, USA], Penicillin/Streptomycin [100U/ml/100 mg/ml, Invitrogen, Carlsbad, CA, USA], R-Spondin 3 [250 ng/ml, R&D Systems, Minneapolis, MN, USA], N-Acetylcysteine [1.25 mM, Sigma, Saint Louis, MO, USA], Noggin [100 ng/ml, Peprotech, Rocky Hill, NJ, USA], Primocin [50 mg/ml, InvivoGen, FGF 10 [20 ng/ml, Peprotech, Rocky Hill, NJ, USA], Neuregulin 1 [5 nM, Peprotech, Rocky Hill, NJ, USA], FGF 7 [5 ng/ml, Peprotech, Rocky Hill, NJ, USA], EGF [5 ng/ml, Peprotech, Rocky Hill, NJ, USA], Y-27632 [5 uM, Abmole, Houston, TX, USA], A83-01 [500 nM, Tocris, Avonmouth, Bristol, UK], SB202190 [500 nM, Sigma, Saint Louis, MO, USA] [13].

The medium was changed every 3 days and passaged every 2 weeks: 1 mL of TrypLE Express (Invitrogen, Carlsbad, CA, USA) was added to the BME-cell suspension droplets, incubation for 20 min at 37 °C, and mechanical blowing. When the number of single cell in the field of view under the microscope reached 90%–95%, the digestion was stopped. 3 mL AdDF+++ was added and centrifugated at 1000 rpm. The pellet was suspended in cold BME and reseeded in the ratio (1:2) as described above.

### Genomic DNA analysis

The tumor tissues and normal oral epithelial cells were used for extraction of genomic DNA respectively, and subjected to whole exon sequencing analysis. Based on second-generation sequencing technology, four types of 1021 tumorigenesis related genes (including point mutations, small fragment insertions, copy number variations, and currently known fusion genes) were detected (Gene^+^, China).

### Drug screen

The organoids were dissociated into individual cells according to the passaging procedure described above and allowed to grow for 7 days. The cell pellet was collected and diluted to approximately 70 organoids/μl in growth medium containing 5% BME. A 384-well plate (Nest, Wuxi, Jiangsu, China) was coated with 10 μL BME, and 30 μL of the suspension was added. Then, 9 concentrations of Tamoxifen, Fulvestrant, Palbociclib, Everolimus, PI3K inhibitor (BKM120) (MedChemExpress, Monmouth Junction, NJ, USA) and DMSO control were added. After 5 days, 25 μL of CellTiter-Glo3D reagent (Promega, Madison, WI, USA) was added to each well and the plate was shaked at room temperature for 30 min. Luminescence was read on a SpectraMax microplate reader (Molecular device). The data was analyzed using GraphPad Prism 6 and the IC_50_ values were determined manually.

## Results

### Case report

**Fig. 1:**
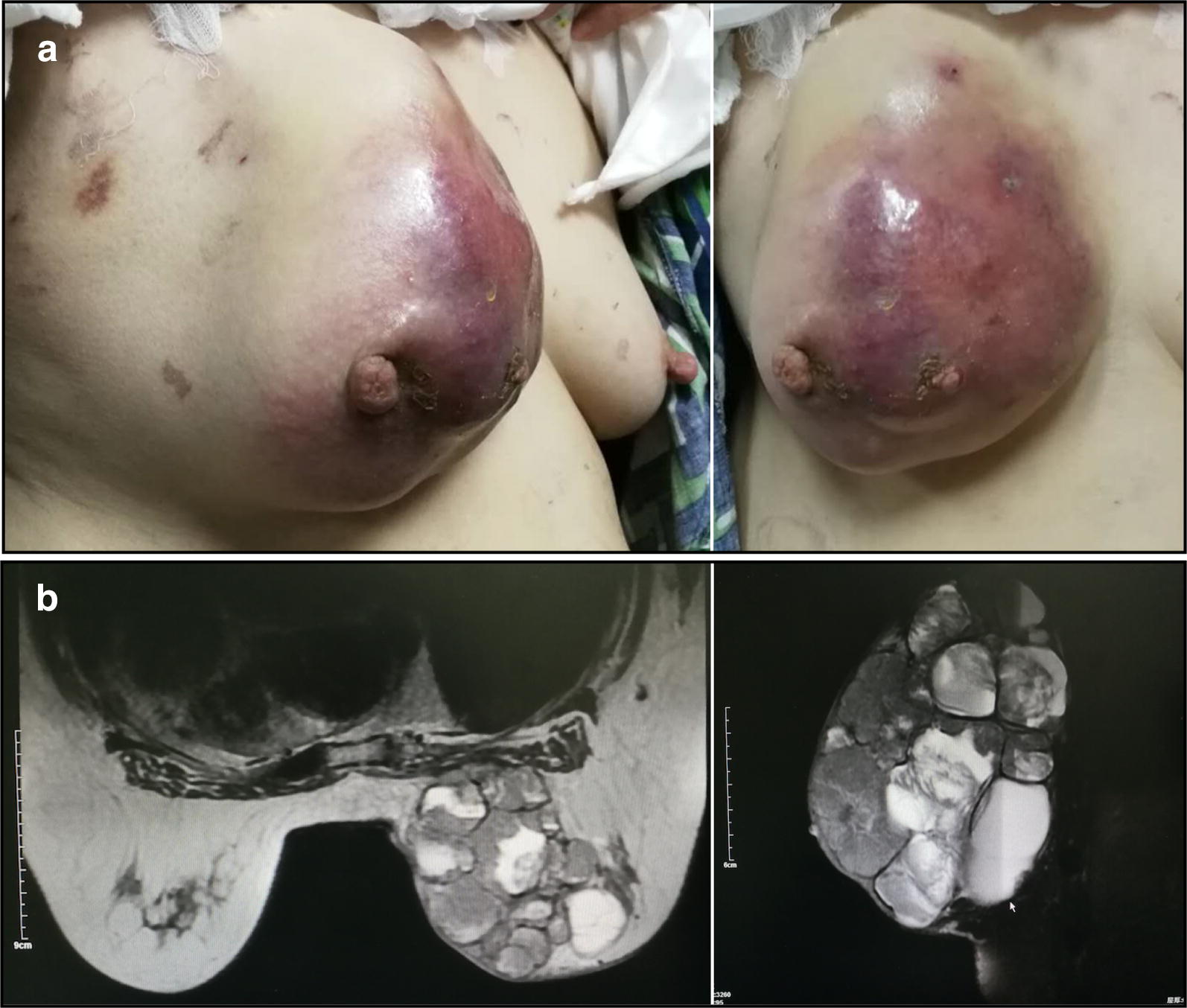
A giant papillary carcinoma of right breast in a 63-year-old Chinese woman was observed by visual examination and MRI scan. **a** The tumor increased rapidly 1 month ago, the skin was red and swollen, and it could touch the size of 12*12 cm hard mass with skin ulceration. **b** Chest MRI showed large cystic and solid mass of irregular shape. The solid part was significantly enhanced after enhancement

A 63-year-old Chinese woman was admitted with a painful, greater than 15 cm mass in her right breast. The patient reported that the lesion had rapidly grown in size within 1 month, and had no family history. Physical examination revealed a hard and unmovable mass with inverted nipple. The medial skin overlying the lesion became suppurated and ulcerated (Fig. 1a). No axillary lymph nodes (LN) were palpable. The contralateral breast appeared normal. The magnetic resonance imaging (MRI) demonstrated that a giant mass occupied almost the entire right breast, showing many large fluid areas with thick septa and a more prominent solid component (Fig. 1b). A whole-body staging examination was negative for axillary LN involvement and distant metastases.

**Fig. 2:**
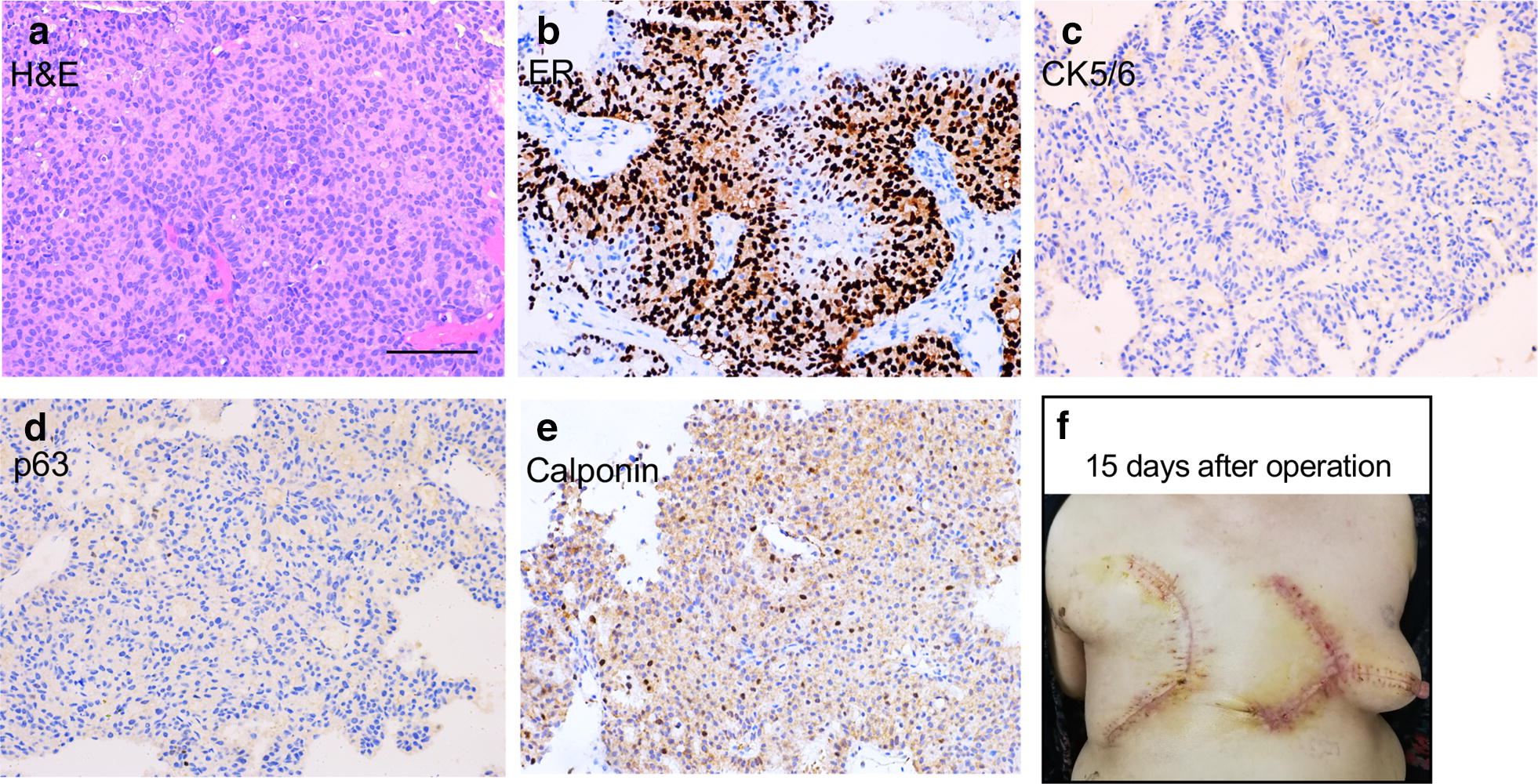
Representative images of preoperative biopsy samples and postoperative recovery from a 63-year-old Chinese woman with giant papillary carcinoma of right breast. **a**–**e** Representative images of H&E and immunohistochemistry staining of preoperative biopsy samples. ER Strongly Positive 90%, CK5/6-, p63-, Calponin-. **f** 15 days after the modified radical mastectomy with a V–Y advancement flap of contralateral breast. Scale bar = 100 μm

A core needle biopsy revealed the papillary structure of tumor cells (Fig. 2a), and IHC confirmed that ER staining was positive (ER Strongly Positive 90%) (Fig. 2b) and myoepithelial cells disappeared (CK5/6-, p63-, Calponin-) (Fig. 2a, d and e). Considering that the lesion rapidly increased within 1 month and was larger than 15 cm, the sentinel lymph node biopsy was not the most suitable option for her situation. Therefore, the modified radical mastectomy was performed with a V–Y advancement flap of contralateral breast to resolve the problem of skin cover in the chest wall (Fig. 2f).

**Fig. 3:**
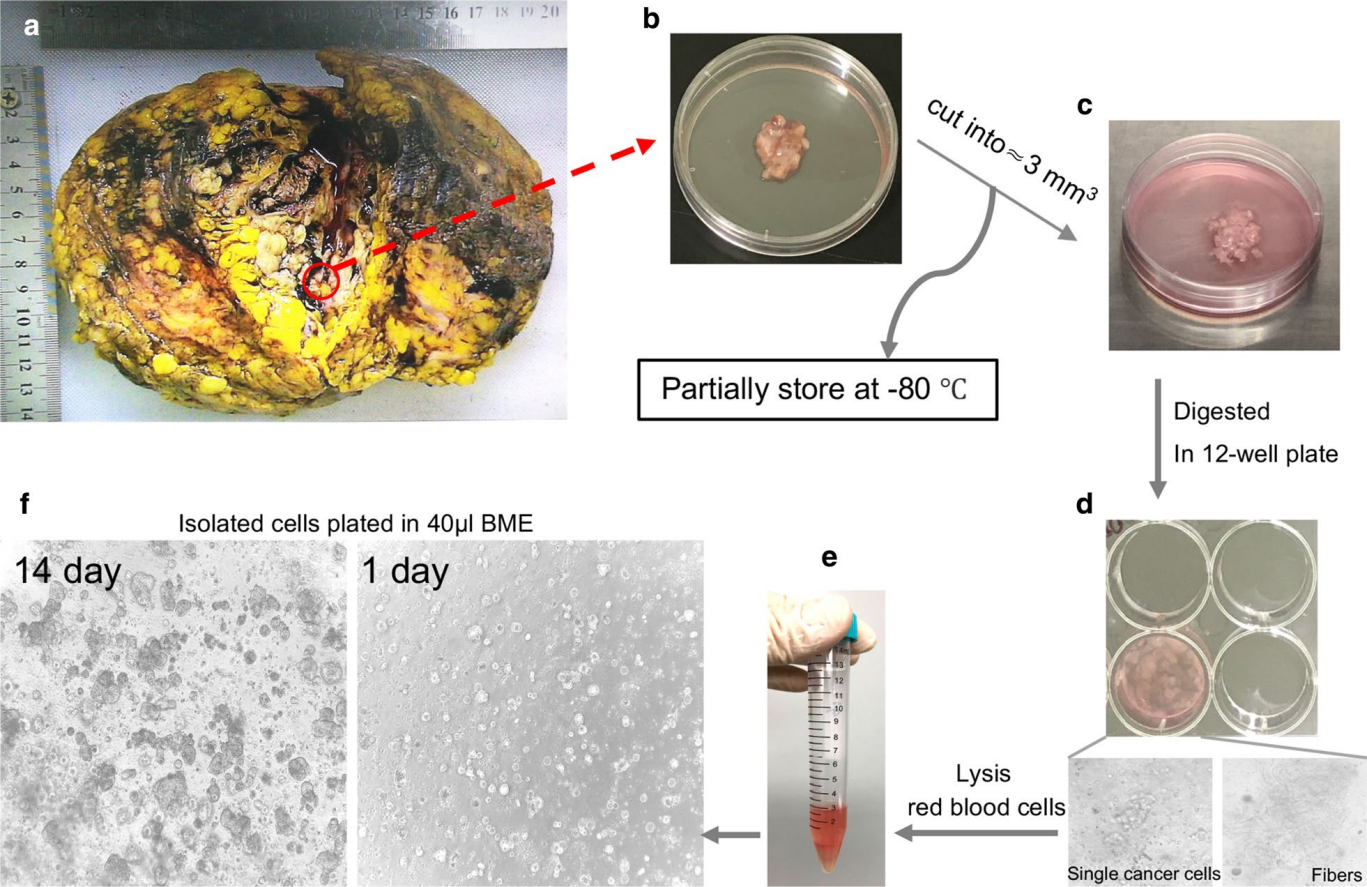
Representative images of the process of organoids culture from a giant papillary carcinoma patient. **a** A giant breast mass surgery specimen. **b** Cut an appropriate size of breast mass for culture and store. **c** The tumor tissue was cut into 3 mm^3^ pieces. **d** You can see the cell mass and fibers when the tumor pieces were digested in collagenase. **e** Lysis red blood cells if a red precipitate is seen. **f** Morphology of organoids under microscope at day 1 and 14

**Fig. 4:**
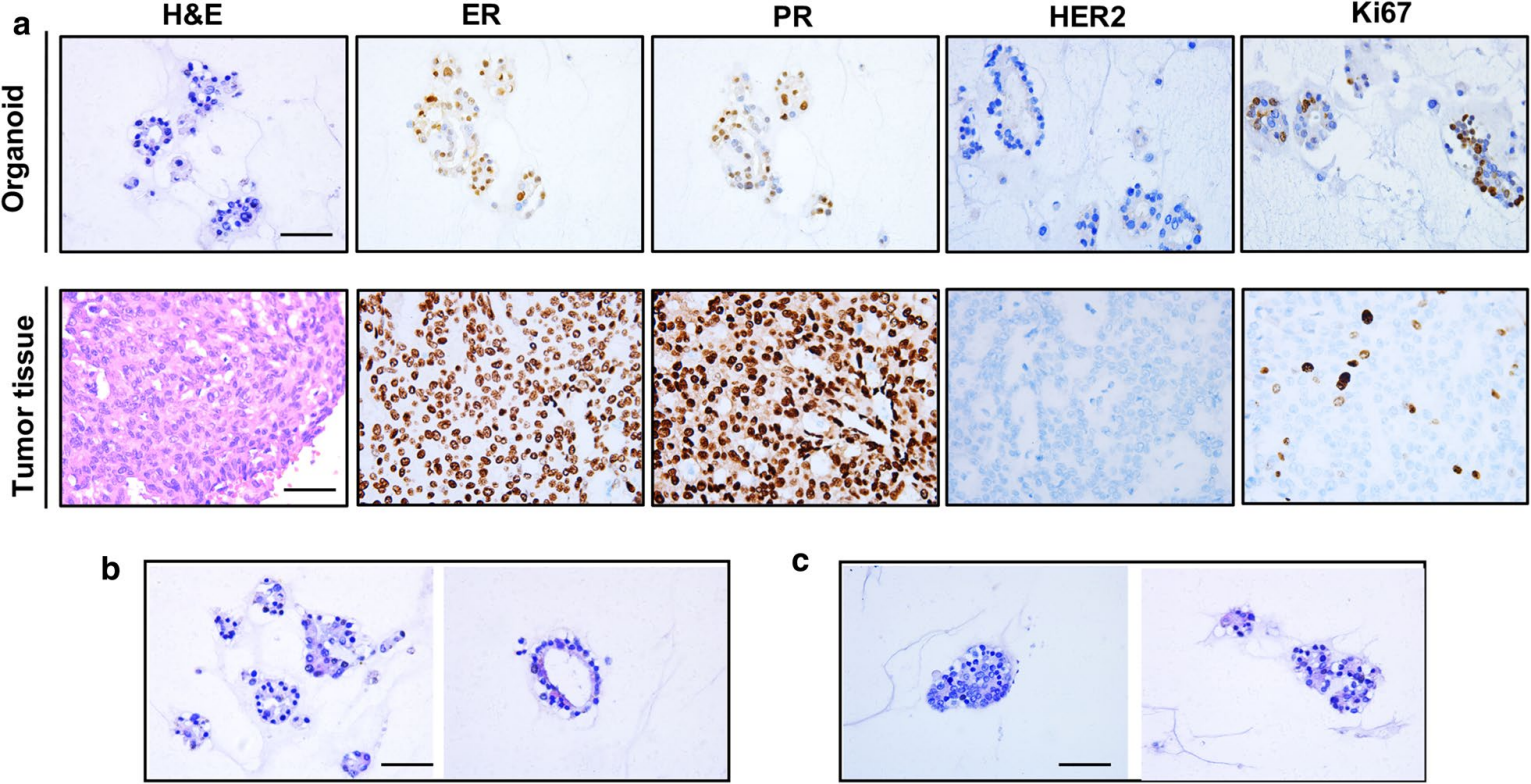
Histopathological characteristics of derived organoids and tumor tissue from papillary carcinoma of breast. Scale bar = 50 μm. **a** Representative images of H&E and immunohistochemistry staining. ER, estrogen receptor; PR, progesterone receptor; HER2, human epidermal growth factor receptor-2; Ki-67, Antigen Ki67. Representative images of layered organoids (**b**) and solid (**c**)

The gross pathologic inspection showed a multi-cavity tumor with expansive and multiloculated growth, and the wall thickness of the cystic part was about 0.1–0.3 cm, filled with stale blood and necrotic debris (Fig. 3a). Postoperative pathology result reported encapsulated papillary carcinoma with intraductal papillary carcinoma without infiltration of the skin and muscle, and axillary LN 0/24. Postoperative IHC pathology further confirmed the high expression of ER and PR (Fig. 4 and Additional file 1: Figure S1). This patient is currently receiving aromatase inhibitor Letrozole. At 1-year follow-up, the patient was free of disease.

### Establishing a papillary carcinoma patient-derived organoid

In order to evaluate the feasibility of primary culture of papillary tumor cells, we used organoid technology to culture these tumor cells of papillary carcinoma from our patient. We obtained breast cancer tissues from this patient who underwent the mastectomy (Fig. 3a). We removed normal tissue visible to the naked eye around the tumor carefully so as not to affect the culture. Subsequently we isolated breast cancer cells through enzymatic digestion (Fig. 3b–d). We recommended to use the collagenase at a concentration of 2 mg/ml in combination with mechanical blowing to shorten digestion time. It could be observed that the tumor cell mass separated from the fiber and fell off under the microscope (Fig. 3d, down panel). With the digestion time increasing, the fibers became viscous. With the prolongation of digestion time and the mechanical blowing, the viscous material would eventually disappear, leaving only the cell mass. Even though red blood cells died after 4–5 days, a large number of red blood cells and subsequent dead red blood cell fragments would still affect the initial culture environment of the organoid. If there was a red precipitation, we recommended to use red blood cell lysis buffer (Fig. 3e). Isolated cells were plated in BME drops and overlaid with organoid culture medium (Fig. 3f). BME drops were the key to maintain excellent growth of organoid. There were two important factors: (1) One was that the concentration of BME could not be too low, and (2) the other was that BME droplets could not perfectly form in the middle of a 24-well plate, partially diffused into the circumference of the dish, which led to uneven droplet distribution. Both of these reasons could cause BME drops to be unstable and broken. Therefore, we recommended to dilute the concentration of BME to 12 mg/ml to keep the droplets stable for a longer time. We slowly and accurately injected 40 μl drops of BME-cell suspension into the center of the dish and kept it stable requiring constant practice. Individual breast cancer organoids were largely different in their morphology. The bright-field morphology of the organoids derived from our papillary carcinoma patient is different from that of previously reported breast cancer patients [13]. We observed solid hard organoids of distinct sizes by bright-field microscopy from a single cell to a larger cell mass (Fig. 3f). When the organoids were cultured for 8 days, it began to enter into the logarithmic growth phase. The number of organoid masses increased rapidly, with the volume increased gradually. After the organoids were cultured for 17 days, they began to enter stagnate phase. It could be found that the growth of the organoids was obviously slow down. We believed that the growth of organoids was related to their intensity. When the organoids were in passage, we suggested single cell suspensions were seeded at high density because the growth of high-dense organoid was far superior to low-density. We successfully established the papillary carcinoma organoids, which had been continuously propagated for more than 6 months.

### Breast cancer organoids match the original histological characteristics

We performed histopathological analysis of H&E stained tissues and organoid sections. We confirmed that the phenotypes of organoids matched the original histological breast cancer types. Besides histological conservation, we also found the papillary carcinoma organoid keeping expression of the breast cancer biomarkers: estrogen receptor (ER), progesterone receptor (PR), human epidermal growth factor receptor (HER2), and antigen KI-67 (Ki67) (Fig. 4a). The status of hormone receptors ER and PR could help us to predict the outcome of endocrine therapy for breast cancer. The analysis of IHC also demonstated strongly positive staining of Ki67 (Fig. 4a). According to the previous report, breast ductal carcinoma usually produced solid and coherent organoids, while breast lobular carcinoma mainly presented discrete organoids [9]. However, we found the organoids from papillary carcinoma were different from the above two pathological types, which produced typical solid and layered organoids (Fig. 4b, c). Based on cellular and nuclear atypia, the papillary carcinoma organoid clearly showed malignant characteristics. The cell size was also distinct and enlarged. The nucleoplasm ratio was inverted. In addition, the nuclear membrane was irregular and the nuclear chromatin was uneven. The coarse particles were similar, and some of the nuclei were vacuolated (Fig. 4c).

In summary, we found that our papillary carcinoma organoid was consistent with the original patient in histopathology and hormone receptor status, which was a high-fidelity model.

### Drug sensitivity test on papillary carcinoma organoid as a personalized therapeutic tool

**Fig. 5:**
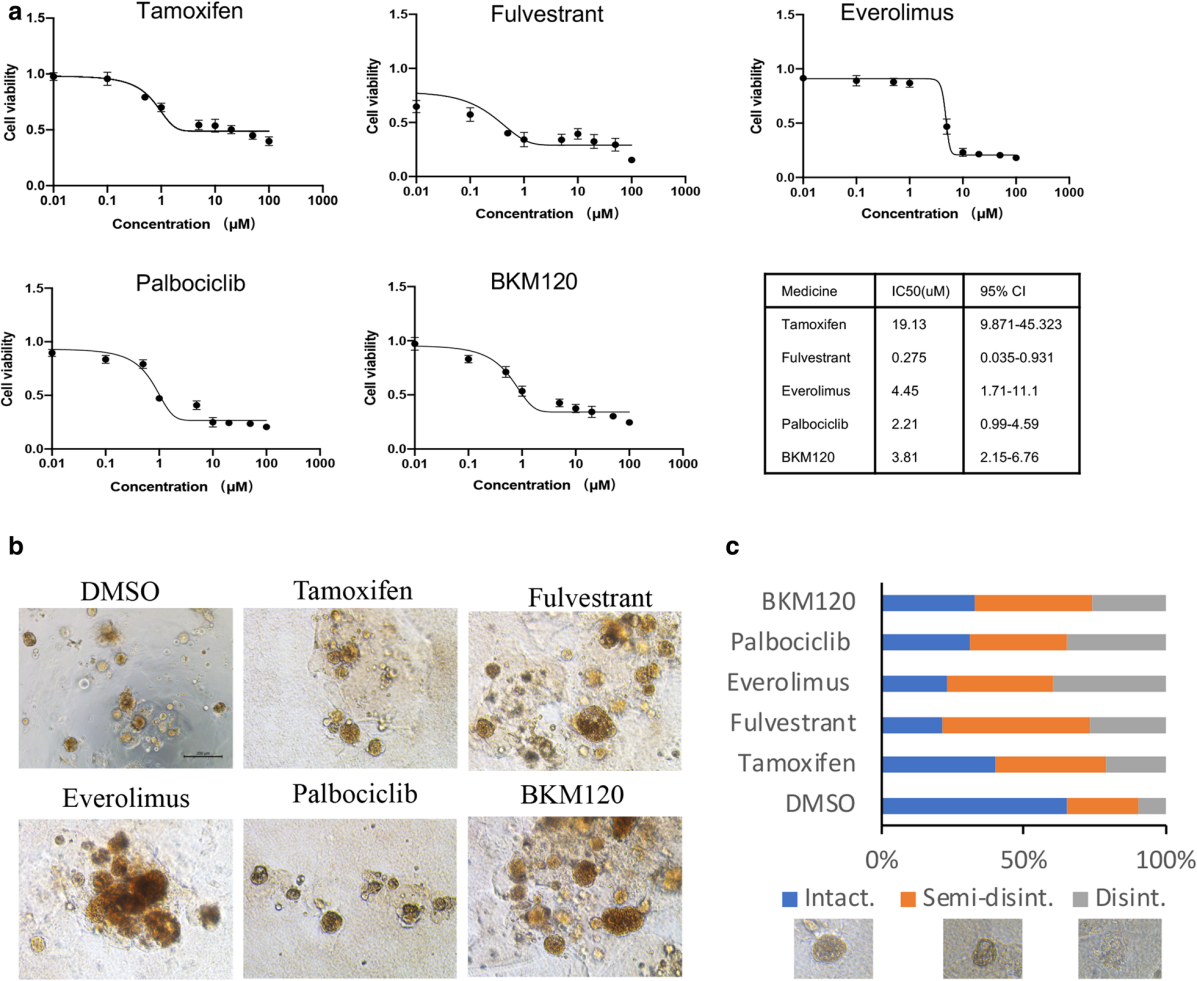
Representative images and drug sensitivity of organoids from a giant papillary carcinoma of right breast in a 63-year-old Chinese woman. Scale bar = 200 μm. **a** Representative images of dose response curves for organoids treated with Tamoxifen, Fulvestrant, Palbociclib, Everolimus, PI3K inhibitor (BKM120). Mean ± SD of results from 3 independent experiments is shown for each drug. **b** Organoids were grown in 3-D culture and treated with indicated drugs. Over 20 structures were scored for each drug. **c** Representative images of scored structures: Intact, Semi-disintegrated, and Disintegrated (Disint.)

Based on the second-generation sequencing, we detected 8 somatic mutations and two out of eight mutations are related to targeted drugs. PTEN p.Y176* mutation and PTEN deletion suggest that the tumor is sensitive to everolimus, with low tumor mutation load (TMB-L, 2.88Muts/Mb, 15%*) and microsatellite stability (MSS) (Additional file 1: Figure S2). In addition, according to the positive staining of ER and PR, we tested the drug sensitivity of cultured organoids to several clinically used endocrine therapy drugs (Tamoxifen, Fulvestrant) and targeted therapy drugs (Palbociclib, Everolimus, BKM120). To mimic the in vivo environment more closely, we performed drug sensitivity experiments in 3D condition. Of all five drugs tested, Fulvestrant (IC_50_ 0.275 μmol, 95% CI 0.035–0.931) showed the best anticancer effect (Fig. 5a). The other regimens only exhibited modest inhibitory effects on tumor cell viability tests (Fig. 5b, c). Altogether, tumor-derived organoids from papillary carcinoma might serve as a predictive model for preclinical assessment of potential effective treatment regimens.

## Discussion

According to the WHO breast tumor classification [22], the pathology of papillary carcinoma of the breast is divided into intraductal papilloma with atypical ductal hyperplasia or DCIS, intraductal papillary carcinoma, coated papillary carcinoma or with infiltration, and solid papillary carcinoma or infiltration. Papillary carcinoma is rare, whose incidence is 0.5% to 1.0% in breast cancer, however, it is more common in postmenopausal women and older women with favorable prognosis [2–4]. This patient is a 63-year-old woman who was admitted with a painful, greater than 15 cm mass in her right breast. All clinicopathological features met the characteristics of this disease.

Organoid culture technique (three-dimensional culture) is different from our previous studys (two-dimensional culture) used in endometrial cancer patients [23] and breast cancer patients with leptomeningeal metastasis [24]. Our cultured ascites-derived tumor cells and cerebrospinal fluid-derived tumor cells gradually underwent senescence after six or seven passages. However, using breast cancer organoid culture technology, the papillary carcinoma organoids could be maintained for a longer time (more than 15 generations, more than 6 months). This relied on the special organoid medium [13] and three-dimensional (3D) culture model, a condition that more closely mimics the in vivo environment. Breast cancer organoid medium allowed the efficient generation of breast cancer organoids as well as their long-term expansion for > 20 passages [13]. Compared with the previous 2D culture, the 3D culture of the organoids mimics the microenvironment of tumor growth more realistically, and the 3D sphere structure is closer to the pathophysiological state of the organ [25]. In addition, long-term organoid cultures were also established from colorectal cancer [14], gastrointestinal cancer [15], pancreas cancer [16], liver cancer [17], prostate cancer [18], bladder cancer [19], and lung cancer [20], with similar success rates. In this study, our breast cancer organoid matched the paired breast cancer tissue with respect to histopathology, hormone receptor statue (ER/PR) and Ki67 index. To our knowledge, this is the first time that such rare tumor-derived organoids from papillary carcinoma have been successfully cultivated, and confirmed high fidelity in histological characteristics.

We tested the drug sensitivity of cultured organoids to several clinically endocrine therapy drugs and targeted therapies. Thses results showed the organoid from breast papillary carcinoma was most sensitive to Fulvestrant single-agent treatment. According to genetic test results, Everolimus was not the most sensitive treatment for our patient. It made us believe that the individualized treatment of patients should be well guided by the combination of gene sequencing technology and drug sensitivity test. Our research revealed Fulvestrant (IC_50_ 0.275 μmol, 95% CI 0.035–0.931) showed the best anticancer effect, but Tamoxifen (IC_50_ 19.13 μmol, 95% CI 9.871–45.323) showed the worst anticancer effect. Consistent with previous in vitro drug experiments, the concentration of Fulvestrant on breast cancer cells might be low, however the concentration of Tamoxifen on breast cancer cells might be high [26, 27]. In clinical applications, the side effects of Tamoxifen are small, thus a higher concentration of Tamoxifen could be used in clinical. Although in vitro experiments of Fulvestrant are indeed good, but the side effects are also particularly large with a slight increase in dosage in vivo experiments that give rise to the poor compliance of the patients.

In addition, we also tried to apply organoids to establish PDX model [28]. We harvested the organoids and injected them into the mammary fat pads or tail intravein of Balb/c Nud mice and NOD/SCID mice at 10^6^ cells in 40 uL of complete breast cancer organoid medium/BME (1:1). However, it is regrettable that the organoids from papillary carcinoma could not form tumors in Balb/c Nud mice and NOD/SCID mice. Compared with ductal carcinoma and lobular carcinoma of the breast, the malignancy degree of papillary carcinoma is considered to be lower, which may result in the failure of establishment of the PDX model. Besides, in the future, we will delineate the molecular pathogenesis and pathophysiology of papillary lesions using this organoid platform.

**Fig. 6:**
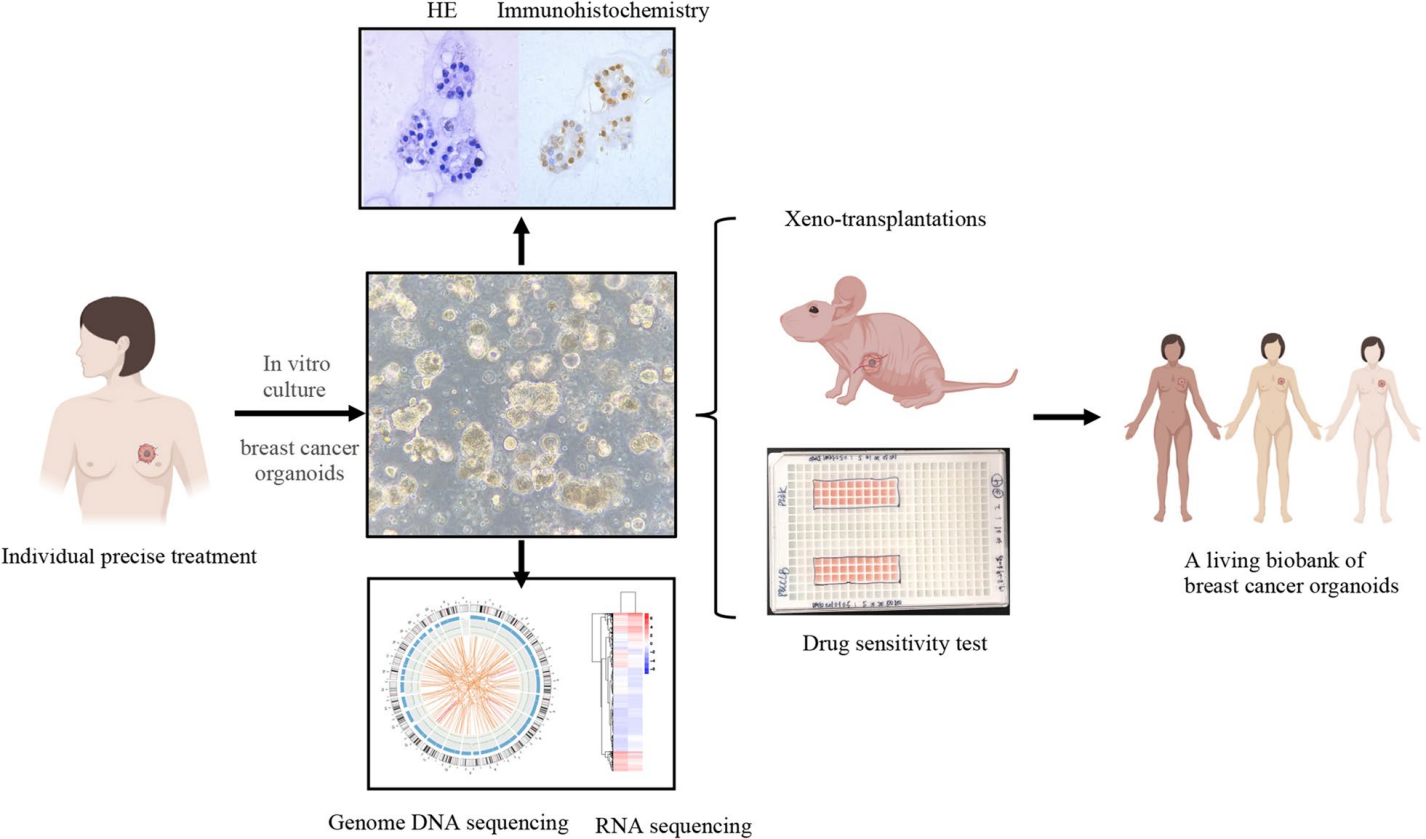
A personalized platform for breast cancer precise treatment. The breast cancer organoids platform can be established from individualized patients. By immunohistochemistry and gene sequencing, the organoids exhibit similar histological and genetic features of original breast tumors. We can use the ex vivo culture of patient-derived organoids and in vivo models to predict potential effective therapeutic protocols. By building a living biobank of breast cancer organoids that comprise the entire spectrum of molecular subtypes per tumor type, we can investigate the etiology and pathological processes associated with breast cancer, promote drug discovery and look for resistance mechanism in the further

To identify therapeutic targets that are likely to be beneficial to an individual patient, we plan to culture patient-derived organoids as a platform (Fig. 6). H&E stain and immunochemistry results prove that organoids match the originating breast cancer with respect to histopathology as well as hormone and HER2 receptor status. By using genome DNA and RNA sequencing, organoids recapitulate the diverse genomic landscape and gene expression profiles of breast cancer. Combine organoids model with PDX model, we can compare in vitro with in vivo drug responses.

## Conclusion

We have successfully established an organoid model that may be a personalized approach to predict potential effective treatment drugs. In addition, we can investigate the etiology and pathological processes associated with breast cancer, thereby promoting drug discovery and exploring resistance mechanism in the further.

## Supplementary information

**Additional file 1: Figure S1.** Histopathological characteristics of tumor tissue from papillary carcinoma of breast. p63-, CK5/6-, Calponin-, EGFR-, p53-. Scale bar = 50 μm.

**Additional file 2: Figure S2.** The second-generation sequencing of the breast cancer tissues. (A) Genomic variation show PTEN p.Y176* mutation and PTEN deletion mutation which suggest the tumor is sensitivity to everolimus. (B) Genomic indicator shows the low tumor mutation load (TMB-L, 2.88Muts/Mb, 15%*) and microsatellite stability (MSS).

## Data Availability

All data during this research are included in this published article.
