# Supplementary figures and images for: Breast cancer organoids from a patient with giant papillary carcinoma as a high-fidelity model
Source: Cancer Cell Int. 2020 Mar 18;20:86. doi: 10.1186/s12935-020-01171-5 (PMC7079375; doi:10.1186/s12935-020-01171-5)

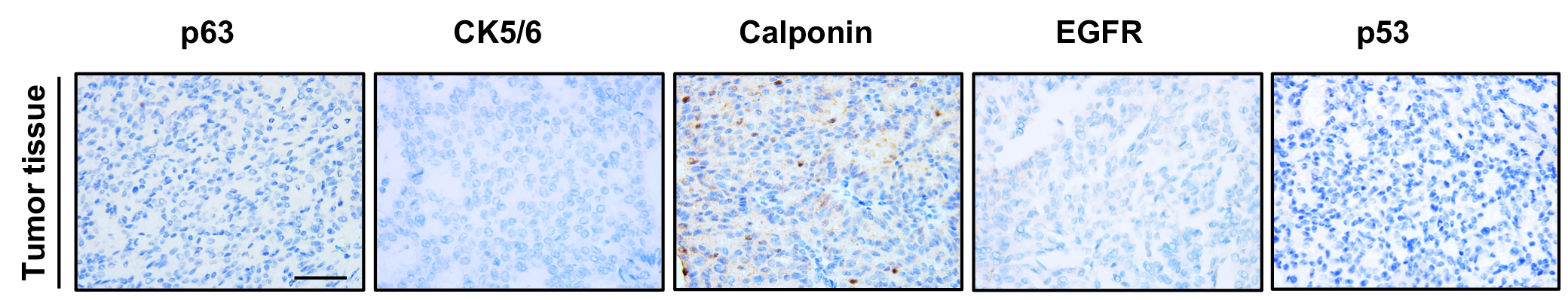

Supplement: Supplementary file 1 — Additional file 1: Figure S1. Histopathological characteristics of tumor tissue from papillary carcinoma of breast. p63-, CK5/6-, Calponin-, EGFR-, p53-. Scale bar = 50 μm. [file 12935_2020_1171_MOESM1_ESM.tif]

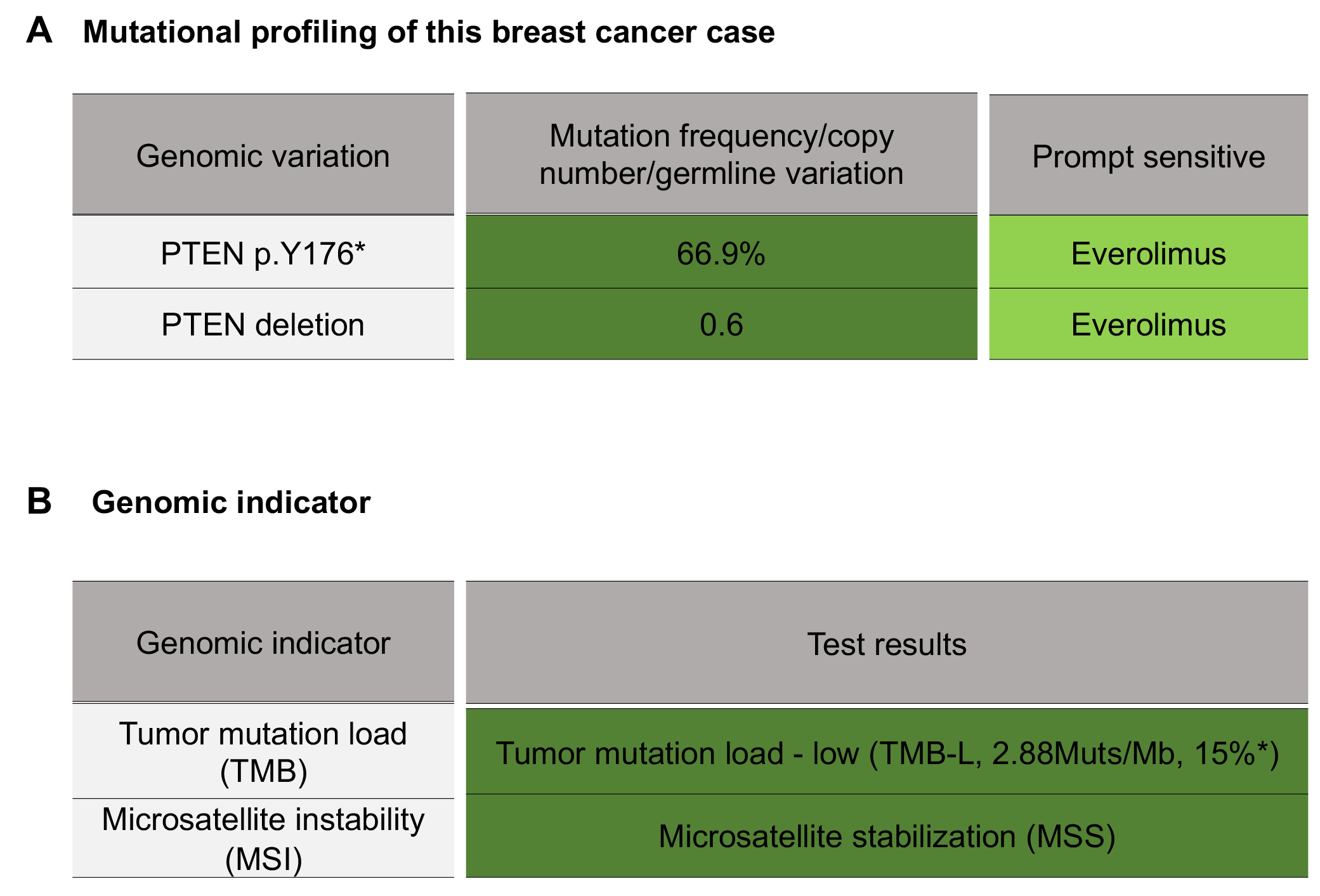

Supplement: Supplementary file 2 — Additional file 2: Figure S2. The second-generation sequencing of the breast cancer tissues. (A) Genomic variation show PTEN p.Y176* mutation and PTEN deletion mutation which suggest the tumor is sensitivity to everolimus. (B) Genomic indicator shows the low tumor mutation load (TMB-L, 2.88Muts/Mb, 15%*) and microsatellite stability (MSS). [file 12935_2020_1171_MOESM2_ESM.tif]
